# A recombinantly tailored β-defensin that displays intensive macropinocytosis-mediated uptake exerting potent efficacy against K-Ras mutant pancreatic cancer

**DOI:** 10.18632/oncotarget.11170

**Published:** 2016-08-10

**Authors:** Yue Du, Bo-yang Shang, Wei-jin Sheng, Sheng-hua Zhang, Yi Li, Qing-fang Miao, Yong-su Zhen

**Affiliations:** ^1^ Institute of Medicinal Biotechnology, Chinese Academy of Medical Sciences and Perking Union Medical College, Beijing, P.R. China

**Keywords:** β-defensin, macropinocytosis, K-Ras mutant, pancreatic cancer, targeting therapy

## Abstract

K-Ras mutant pancreatic cancer cells display intensive macropinocytosis, indicating that this process may be exploited in the design of anticancer targeted therapies. In this study, we constructed a macropinocytosis-oriented recombinantly tailored defensin (DF-HSA) which consists of human β-defensin-2 (DF) and human serum albumin (HSA). The macropinocytosis intensity and cytotoxicity of DF-HSA were investigated in K-Ras mutant MIA PaCa-2 cells and wild-type BxPC-3 cells. As found, the DF-HSA uptake in MIA PaCa-2 cells was much higher than that in wild-type BxPC-3 cells. Correspondingly, the cytotoxicity of DF-HSA to MIA PaCa-2 cells was more potent than that to BxPC-3 cells. In addition, the cytotoxicity of DF-HSA was much stronger than that of β-defensin HBD2. DF-HSA suppressed cancer cell proliferation and induced mitochondrial pathway apoptosis. Notably, DF-HSA significantly inhibited the growth of human pancreatic carcinoma MIA PaCa-2 xenograft in athymic mice at well tolerated dose. By *in vivo* imaging, DF-HSA displayed a prominent accumulation in the tumor. The study indicates that the recombinantly tailored β-defensin can intensively enter into the K-Ras mutant pancreatic cancer cells through macropinocytosis-mediated process and exert potent therapeutic efficacy against the pancreatic carcinoma xenograft. The novel format of β-defensin may play an active role in macropinocytosis-mediated targeting therapy.

## INTRODUCTION

Macropinocytosis is a highly conserved endocytic process by which extracellular fluid and its contents are internalized into cells through large, heterogeneous vesicles known as macropinosomes [1]. Macropinocytic vesicles have no apparent coat structures and the size is considerably larger than the clathrin coated vesicles. Owing to the large size of macropinosomes, the process provides cells with a fast and effective way to internalize extracellular nutrients and solute molecules [2]. Most cells do not exhibit macropinocytic activity under normal culture conditions, while macropinocytosis widely exists in tumor cells; it is an important nutrient delivery pathway that cancer cells use to drive their proliferation and growth [3, 4]. In cancer cells, the oncogenic expressions, such as Ras and Src, exhibit intensive macropinocytosis [3, 5]. In addition, the stimulation of cancer-related receptors, such as the epidermal growth factor receptor (EGFR), can induce the generation of macropinocytosis [6]. Most of the lung cancer, colon cancer, and more than 90% of the pancreatic cancer are K-Ras mutated [7, 8]. As reported, K-Ras-transformed cells intensely depend on enhanced macropinocytosis to transport extracellular proteins into the cells for maintaining their proliferation and growth. The internalized proteins undergoes proteolytic degradation to produce essential amino acids to support their unique nutritional needs [1, 9]. Blocking the macropinocytosis-mediated protein uptake will render tumor cells stopping proliferation. Therefore, modulation of the macropinocytosis process might serve as a promising strategy for the development of novel cancer targeted therapeutics. Based on the macropinocytosis modulation approach, the newly designed antitumor therapeutics might improve selectivity and delivery efficiency. Recent study has revealed that oncogene-stimulated intensive macropinocytosis is an entry route for extracellular albumin [3]. Accordingly, it is feasible to employ albumin as a carrier for macropinocytosis-mediated targeting therapy.

Human defensins, acting as an important component of the innate immune system, are small cationic peptides stored in cytoplasmic granules of neutrophils, macrophages, and certain types of epithelial cells; and generally, they are released into extracellular environment [10]. Defensins can be divided into two subgroups, α- and β-defensins, based on both the unique amino acid sequences and the disulfide connectivity [11, 12]. Defensins play important roles in innate immune defense, through neutralizing bacterial toxins or disrupting the cytoplasmic membrane to kill microbial pathogens [13, 14]. Additionally, they may also involve in adaptive immunity by serving as chemoattractants and activators of the immune cells [15, 16]. Previous studies have shown that human defensins have antibacterial, anti-viral activity and tumor cell cytotoxicity. Compared with traditional chemotherapeutics, defensins may have the advantage that it is difficult for cancer cells to develop drug resistance; in addition, they show no immunogenicity and are resistant to proteolysis [10, 17]. Apparently, defensin peptides might be potentially useful in cancer treatment [16–18].

Human β-defensin-2 (HBD2) is an inducible antimicrobial peptide [19]. In addition, it exhibits cytolytic activity against a range of tumor cells that is thought either to destabilize the cellular membrane or to cause the formation of open pores which allow vital biomolecules leaking out of the cell. Consequently, the cells will burst and die [20]. It is the drastic destruction effect of defensin on the cellular membrane that makes cancer cells more difficult to develop resistance [17]. By this action, HBD2 could execute the killing of cancer cells [21–24]. Studies revealed that HBD2 expression was significantly lower in tumors compared with normal tissues [10, 25, 26]. In vitro study, HBD2 at rather high concentrations exerts significant concentration-dependent growth suppression of various cancer cells [21–24]. Furthermore, genetic therapy with mouse β-defensin 2 inhibits tumor growth of lung carcinoma and sarcoma in mice [27]. Evidently, β-defensin 2, a major mediator of innate immunity, has a potential application in cancer therapy.

Considering the fact that intensive macropinocytosis plays an essential role in a variety of cancers, in particular the K-Ras mutant pancreatic cancer; and defensins, in particular HBD2, may play an active role in suppressing tumor growth, the present study is set to construct a macropinocytosis-oriented tailor-made defensins, including HBD2-HSA (DF-HSA) and HSA-HBD2 (HSA-DF), which consist of the polypeptide HBD2 and human serum albumin (HSA) by DNA recombination generated through the *Pichia pastoris* expression system, to determine the intensive macropinocytosis-mediated intracellular entry in pancreatic carcinoma cells, to assess the inbound defensin-related cytotoxicity, and to evaluate the therapeutic efficacy of the albumin-integrated defensin DF-HSA in pancreatic carcinoma xenograft in athymic mice. The study provides evidence that the albumin-integrated defensin bestowed with intensive macropinocytosis attribute is highly effective against K-Ras mutant pancreatic cancer.

## RESULTS

### Construction, preparation and characterization of albumin-integrated defensins

**Figure 1:**
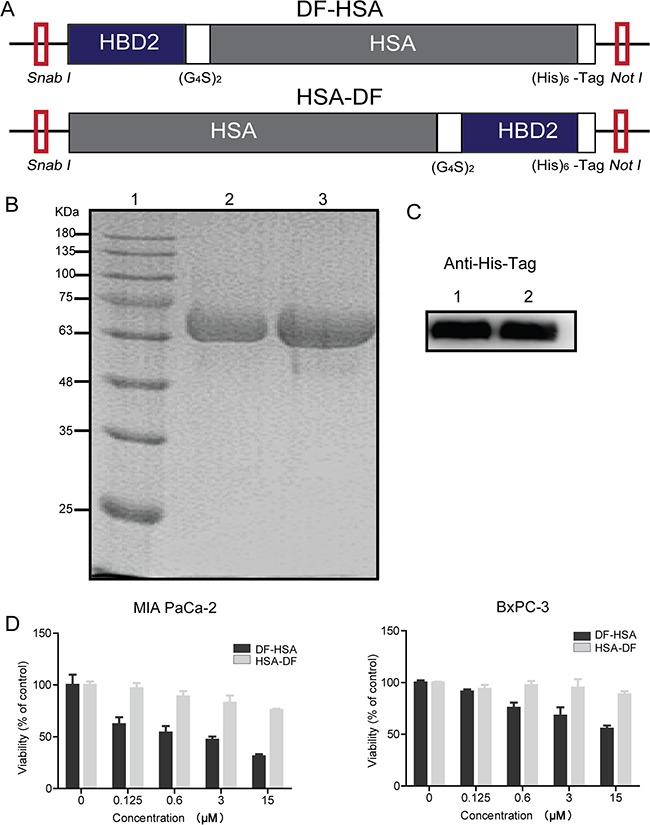
Construction, expression and cytotoxicity of the albumin-integrated defensins **A.** Schematic diagram of *SnaB I/Not I* gene fragments encoding for the albumin-integrated defensin DF-HSA (upper row) and HSA-DF (lower row), respectively. **B.** Purity analysis of the proteins DF-HSA and HSA-DF by 10% SDS-PAGE under denaturing conditions. Lane 1, molecular weight marker; Lane 2, the purified HSA-DF; Lane 3, the purified DF-HSA. **C.** Western blot detection of the proteins DF-HSA and HSA-DF using mouse anti-His tag monoclonal antibody (1/2000 dilution) and HRP-conjugated goat anti-mouse IgG (1/5000 dilution). Lane 1, the purified HSA-DF; Lane 2, the purified DF-HSA. **D.** Cytotoxicity of DF-HSA and HSA-DF respectively to MIA PaCa-2 and BxPC-3 pancreatic carcinoma cells as determined by MTT assay.

The DNA fragments encoding for the human serum albumin integrated defensins DF-HSA and HSA-DF were obtained by genetic engineering, as shown in Figure 1A. The engineered proteins were successfully expressed in *Pichia pastoris* and secreted into the culture in a soluble form with a six-histidine tag at the carboxyl-terminus. The purity of fusion proteins was analyzed by 10% SDS-PAGE and Western blot, as presented in Figure 1B and 1C, the purified proteins migrated as a single band at approximately 72 kDa and the purity of both proteins was over 90%. The final yield of DF-HSA and HSA-DF was 20 and 25 mg/L, respectively.

Cytotoxicity of these two proteins DF-HSA and HSA-DF was tested in MIA PaCa-2 and BxPC-3 pancreatic carcinoma cells, respectively. As shown in Figure 1D, the protein DF-HSA that the defensin attaches to N-terminal of albumin was more potent than HSA-DF in which the defensin attaches to C-terminal of albumin; in particular, the difference was more significant in the K-Ras mutant MIA PaCa-2 cells. This may be related to the protein structure and the steric hindrance. On the basis of this finding, we focused on DF-HSA in the following studies.

### Visualization and quantification of the macropinocytosis-mediated uptake of DF-HSA in pancreatic cancer cells

**Figure 2:**
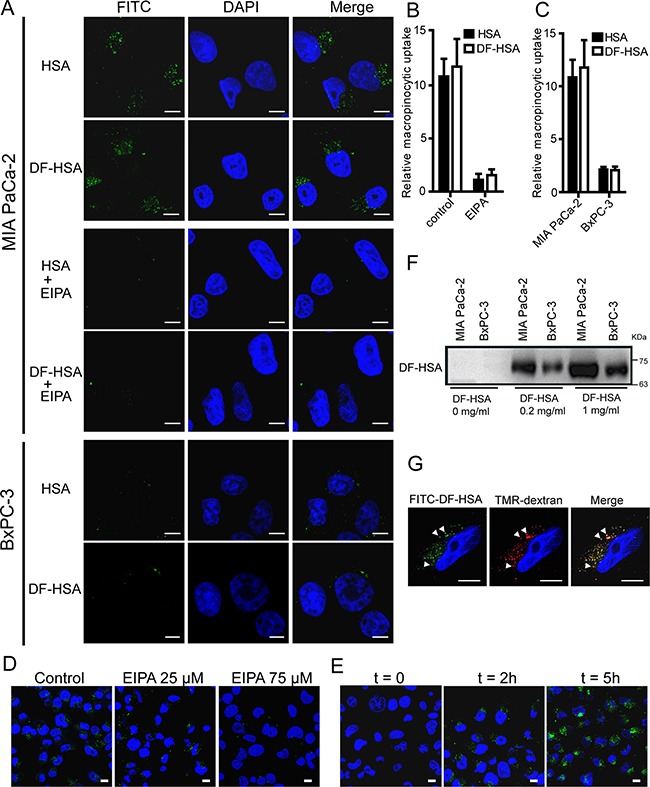
DF-HSA uptake by pancreatic carcinoma cells **A.** Confocal microscopic observation of MIA PaCa-2 and BxPC-3 cells treated with FITC-HSA and FITC-DF-HSA at 37°C for 2 h, and the difference in MIA PaCa-2 cells treated with 75 μM EIPA or not. **B.** Quantification of macropinocytic uptake in MIA PaCa-2 cells treated with 0 or 75 μM EIPA. Data are presented as arbitrary units relative to the values obtained in 75 μM EIPA condition. **C.** Quantification of macropinocytic uptake in MIA PaCa-2 and BxPC-3 cells. Data are presented as arbitrary units relative to the values obtained for BxPC-3 cells. **D.** Confocal microscopic observation on the macropinocytic uptake of FITC-DF-HSA in MIA PaCa-2 cells treated with 0, 25, and 75 μM EIPA, respectively. **E.** Analysis of FITC-DF-HSA fluorescence in MIA PaCa-2 cells exposed to FITC-DF-HSA for different time intervals (t = 0, 2 and 5 h, respectively). **F.** Western blot detection of the macropinocytic uptake of different concentrations of DF-HSA in MIA PaCa-2 and BxPC-3 cells using goat anti-HBD2 polyclonal antibody (1/200 dilution) and HRP-conjugated rabbit anti-goat IgG (1/5000 dilution). The cells exposed to DF-HSA in different concentrations (0, 0.2 and 1 mg/ml, respectively) at 37°C for 2 h. **G.** FITC-DF-HSA is internalized into discrete puncta in MIA PaCa-2 cells that co-localize (white arrowheads) with TMR-dextran. Green signals, FITC-DF-HSA and FITC-HSA; red signals, TMR-dextran; blue signals, DAPI staining identifies nuclei. For all graphs, error bars indicate mean ± SD for n = 3 independent experiments with at least 200 cells scored per experiment. Scale bar: 10 μm.

The uptake of DF-HSA in pancreatic cancer cells was detected by using laser scanning confocal microscope and Western blot. As shown in Figure 2A and 2B, both HSA and DF-HSA displayed intensive uptake in the K-Ras mutant MIA PaCa-2 cells; and clearly, the uptake was blocked by the addition of EIPA, 5-(N-ethyl-N-isopropyl) amiloride, a macropinocytosis-specific inhibitor. Moreover, FITC-DF-HSA was incorporated into discrete intracellular structures that co-localized with the 70-kDa-molecular-weight TMR-dextran (Figure 2G) which can be selectively internalized into macropinosomes and act as an established marker of macropinocytosis [28]. Evidently, the massive entry of DF-HSA into MIA PaCa-2 cells was macropinocytosis-mediated in nature. Notably, confocal-based uptake analysis indicated that the K-Ras mutant MIA PaCa-2 cells showed a much higher level of FITC-labelled DF-HSA uptake compared with wild-type K-Ras-expressing BxPC-3 cells, which was similar to the uptake level of albumin (Figures 2A and 2C). Furthermore, Western blot results showed that the concentration of DF-HSA protein in MIA PaCa-2 cells was higher than that in BxPC-3 cells when the cells were incubated with DF-HSA (Figure 2F). The inhibition of DF-HSA uptake by EIPA was shown in a concentration-dependent manner (Figure 2D). In addition, there was a time-dependent manner in DF-HSA uptake; with the extension of time, much more DF-HSA entered into MIA PaCa-2 cells with more intense fluorescence (Figure 2E). These results suggest that β-defensin has been successfully brought into cancer cells through macropinocytosis in a molecular form of albumin-integrated defensin. In addition, there existed an intensive macropinocytosis-mediated intracellular entry of DF-HSA in MIA PaCa-2 cells, which was much higher than that in BxPC-3 cells.

### Cytotoxicity of the albumin-integrated defensin DF-HSA to cancer cells

**Figure 3:**
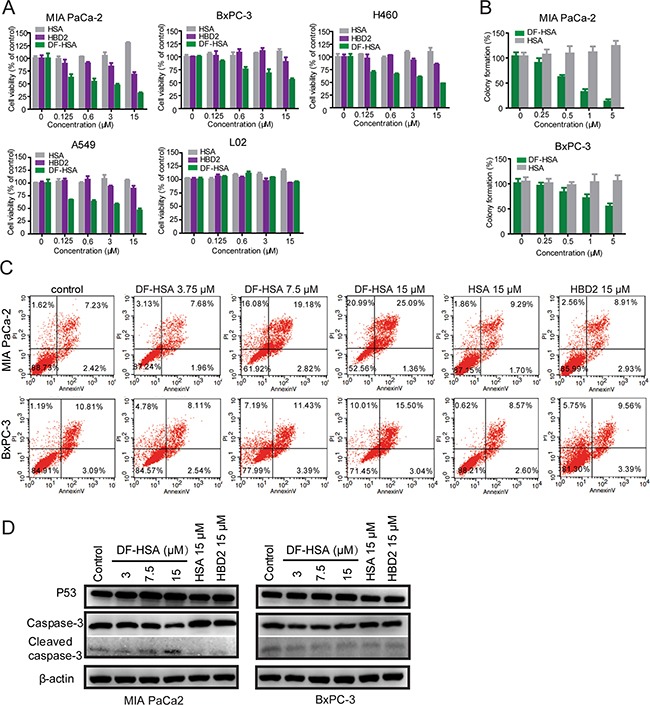
Cytotoxicity of the albumin-integrated defensin DF-HSA to various cancer cells **A.** Growth inhibition analyses of DF-HSA, free HBD2, and HSA to MIA PaCa-2, BxPC-3, A549, H460 and L02 cells, determined by the MTT assay. DF-HSA inhibited cancer cells growth in a concentration-dependent manner. **B.** The effect of DF-HSA and HSA on colony formation capability of MIA PaCa-2 and BxPC-3 cells was determined using clonogenic assay. **C.** Flow cytometry analysis of apoptosis in MIA PaCa-2 and BxPC-3 cells treated with DF-HSA, HBD2, and HSA, respectively. **D.** Western blot showed the levels of apoptosis associated proteins including p53, caspase-3 and cleaved caspase-3 in MIA PaCa-2 and BxPC-3 cells.

The cytotoxicity of DF-HSA was tested with MTT assay in four human carcinoma cell lines including two human pancreatic carcinoma cell lines MIA PaCa-2 and BxPC-3, and two human lung carcinoma cell lines A549 and H460. The cytotoxicity to non-cancerous cells was examined with human hepatocyte cell line L02. HSA and free HBD2 were also tested for comparison. As shown in Figure 3A, HSA slightly promoted the proliferation of cancer cells. By contrast, DF-HSA markedly inhibited cancer cell proliferation. Compared at equivalent concentration, DF-HSA was more potent than free HBD2, suggesting that the potency of defensin was increased in cancer cells by tethering with HSA. As shown, DF-HSA inhibited the viability of MIA PaCa-2, BxPC-3, A549 and H460 cells in a concentration-dependent manner and the IC50 values were 1 ± 0.08, 18.7 ± 1.21, 10.3 ± 0.51 and 12 ± 0.93 μM, respectively. And the IC50 values of free HBD2 were > 100 μM. No cytotoxicity to L02 cells was found at the tested concentrations of DF-HSA. In terms of IC50 values MIA PaCa-2 cells were more sensitive to DF-HSA than BxPC-3 cells; furthermore, DF-HSA was more potent than free HBD2 in equivalent concentrations, implying that the enhanced potency of DF-HSA can be attributed to the intensive macropinocytosis in the K-Ras mutant MIA PaCa-2 cells. Similar results were found with clonogenic assay, DF-HSA showed more significant inhibitory effects on the colony formation capability of MIA PaCa-2 than that of BxPC-3 cells (Figure 3B). Referred to the uptake assay results of DF-HSA in pancreatic cancer cells, there existed an intensive macropinocytosis-mediated intracellular entry of DF-HSA in MIA PaCa-2 cells, which was much higher than that in BxPC-3 cells, these indicate that the inhibitory effect of DF-HSA is a macropinocytosis-mediated action of the albumin-integrated β-defensin remained active while being brought into the cell.

Flow cytometry analysis indicated that the ratio of necrosis and late-stage apoptosis of MIA PaCa2 cells treated with 15 μM DF-HSA was 46.08%; DF-HSA was far more effective than that of free HBD2 (Figure 3C). Much differently, DF-HSA induced only lower levels of apoptosis and necrosis in BxPC-3 cells (25.51%).

Western blot analysis was performed to evaluate the effect of DF-HSA on the expression of apoptosis associated proteins involved caspase-3 and p53. As shown in Figure 3D, compared with the control, there was no significant difference in the expression levels of P53 and caspase-3 in BxPC-3 cells treated respectively with 15 μM HSA, 15 μM free HBD2, and different concentrations of DF-HSA. A similar result was observed in MIA PaCa-2 cells exposed to HSA and HBD2. However, in the case of DF-HSA, the expression level of p53 was upregulated; and procaspase-3 was proteolytically cleaved to produce caspase-3 in concentration-dependent manner. It is indicated that DF-HSA can induce MIA PaCa-2 cells apoptosis through the raising of P53 expression and activation of caspase-3.

### Effects of DF-HSA on the ultrastructure of MIA PaCa-2 cells

**Figure 4:**
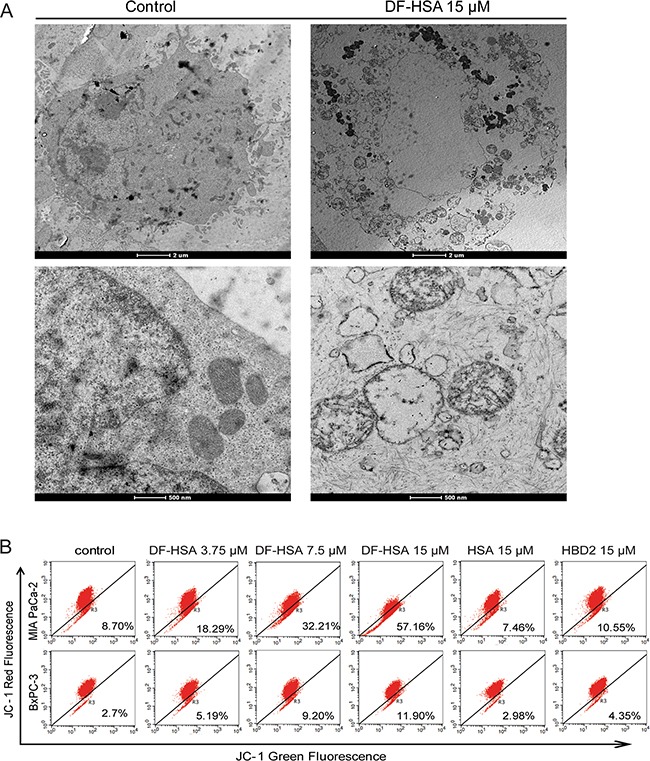
Effects of DF-HSA on ultrastructure and mitochondrial membrane potential in human pancreatic carcinoma cells **A.** Morphological observation with transmission electron microscopy in MIA PaCa-2 cell treated with 15 μM DF-HSA and the control. Upper row: scale bar, 2 μm; under row: scale bar, 500 nm. As shown, mitochondrial changes were found in the treated cells. **B.** Mitochondrial membrane potential of pancreatic carcinoma cells treated with HSA, HBD2 and different concentrations of DF-HSA was assessed by JC-1 fluorescent dye using flow cytometry analysis. The increment of green fluorescence (JC-1 monomer) and the decrease of red fluorescence (JC-1 aggregate) indicated the loss of mitochondria membrane potential in treated cells.

According to the above-mentioned results, a large amounts of DF-HSA was internalized into MIA PaCa-2 cells through macropinocytosis; consequently, exerted potent cytotoxicity to the cells. For further investigation of the subcellular effects of DF-HSA on MIA PaCa-2 cells, transmission electron microscopy was performed to observe the ultrastructural changes in the treated cell, compared with control. As shown in Figure 4A, cell swelling and vacuole formation appeared in the treated cells. With higher magnification, marked mitochondrial changes were observed in DF-HSA treated cells, including mitochondrial swelling, rounded shape in appearance, vacuole formation as well as the separation and disruption of cristae. By contrast, no mitochondrial changes were detected in the untreated control cells. This result indicated that DF-HSA can induce mitochondrial damage.

### Effects of DF-HSA on the permeability of mitochondrial membrane in human pancreatic carcinoma cells

In order to further study the effect of DF-HSA on mitochondria, JC-1 fluorescence dye was used to evaluate the permeability of mitochondrial membrane in DF-HSA treated pancreatic cancer cells. As shown in Figure 4B, after treated with DF-HSA, the intensity of green fluorescence in MIA PaCa-2 cells was increased, while the intensity of red fluorescence decreased. DF-HSA at 15 μM significantly elevated the green fluorescence from 8.7% to 57.17% compared with control. Under the same conditions, the green fluorescence in BxPC-3 cells increased only from 2.7% to 11.9%. By contrast, the fluorescence intensity showed no appreciably changes in MIA PaCa-2 and BxPC-3 cells when treated with HSA and HBD2, respectively. The results indicate that DF-HSA contributed to reduce the mitochondrial membrane potential and the effect was much stronger in MIA PaCa-2 cells than that in BxPC-3 cells.

**Figure 5:**
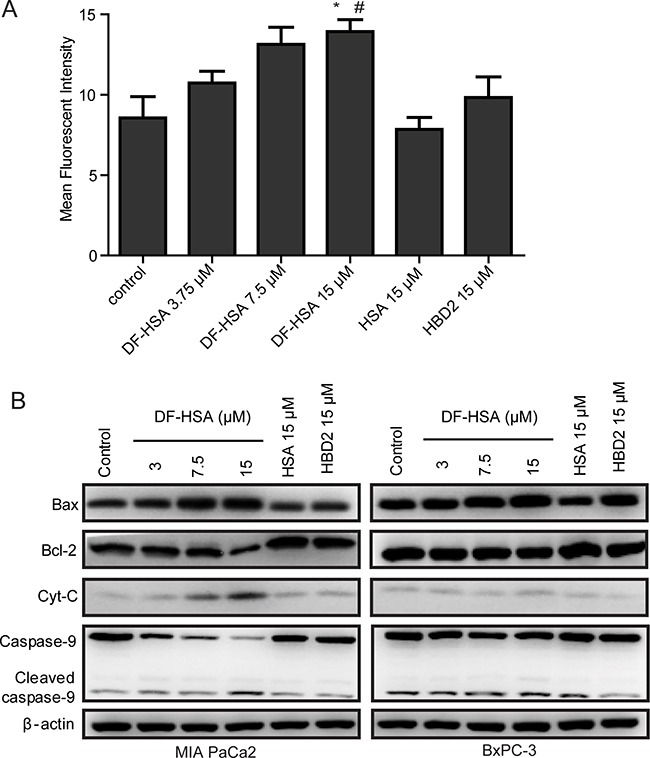
Effects of DF-HSA on the level of ROS and mitochondrial apoptosis pathway related proteins in human pancreatic carcinoma cells **A.** Level of ROS in MIA PaCa-2 cell treated with HSA, HBD2 and different concentrations of DF-HSA was determined using CM-H2DCFDA assay by flow cytometry. *, P ≤ 0.05, compared with the control; #, P ≤ 0.05, compared with the HBD2 group. **B.** Western blot showed the expression level of mitochondrial apoptosis pathway related proteins in MIA PaCa-2 and BxPC-3 cell treated with 15 μM HSA, 15 μM HBD2 and different concentrations of DF-HSA.

As reported, mitochondria are the main cellular source of reactive oxygen species (ROS) [29], and the generation of ROS contributes to the induction of apoptosis in cancer cells [30]. To determine whether the cytotoxicity effect of DF-HSA on human pancreatic carcinoma cells were related to the generation of ROS, fluorescent probe CM-H2DCFDA was used to measure the formation of intracellular ROS in MIA PaCa-2 cells treated with DF-HSA. As shown in Figure 5A, exposure of MIA PaCa-2 cells to 15 μM DF-HSA significantly increased the formation of ROS by 1.62-fold compared to the untreated control (P<0.05). And the relative fluorescence value varied with the doses. In contrast, there was no appreciable increase of ROS when cells treated respectively with HSA and HBD2, indicating that DF-HSA could increase the levels of intracellular ROS in MIA PaCa-2 cells.

To further study the relationship between the induction of apoptosis and mitochondrial changes in pancreatic carcinoma cells, we examined the effect of DF-HSA on the mitochondrial pathway using Western blot. As shown in Figure 5B, DF-HSA and HBD2 had little effect on the expression levels of a mitochondrial pathway proteins in BxPC-3 cells; same to the influence of HBD2 in MIA PaCa-2 cells. HSA did not affect the expression of apoptosis related proteins. By contrast, DF-HSA promoted caspase-9 as well as caspase-3 (reported above). Moreover, treatment with DF-HSA leaded to a significant increase in the levels of cytochrome C, which plays a critical role in activating caspase-3 and caspase-9 and the subsequent mitochondria-mediated apoptosis. In addition, DF-HSA caused an increase in pro-apoptotic Bax protein levels and a decrease in anti-apoptotic Bcl-2 levels compared to the control group. The densitometry data demonstrated that DF-HSA could induce apoptosis of MIA PaCa-2 cells through the mitochondrial apoptosis pathway.

### *In vivo* and *ex vivo* imaging of DF-HSA

**Figure 6:**
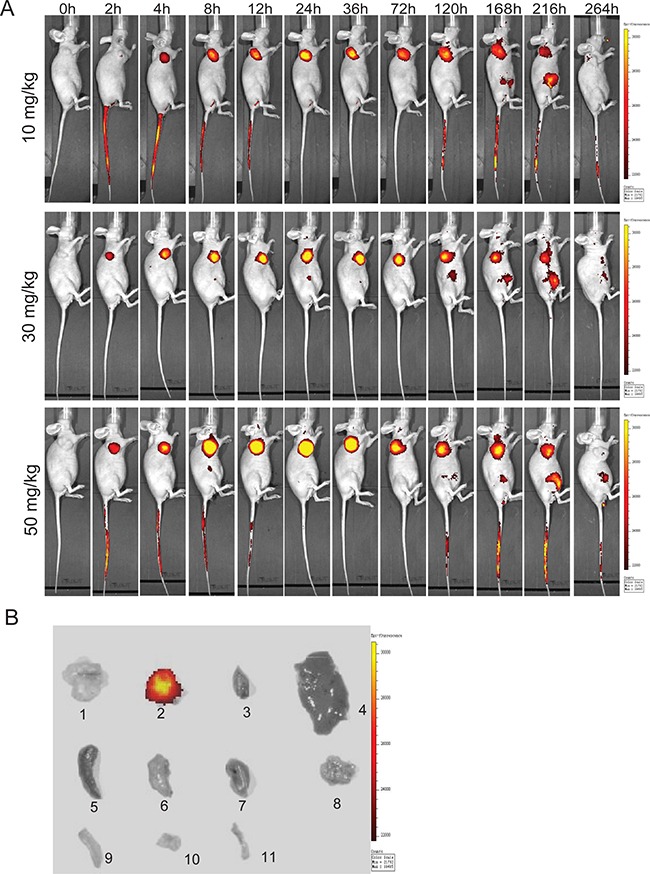
*In vivo* and *ex vivo* optical imaging in MIA PaCa-2 xenograft-bearing athymic mice using Dylight680-labeled DF-HSA **A.** Representative *in vivo* fluorescence images at appointed times after tail vein injection of 10, 30 and 50 mg/kg DF-HSA. Color scale represents photons/s/cm2/steradian. **B.** *Ex vivo* fluorescence images, 1 was the excised tumor from untreated mouse; 2-11 were excised tumor, heart, liver, spleen, lung, kidney, pancreas, large intestine, small intestine and bone from the upper mouse in (A) at 120h.

An optical molecular imaging system was used to evaluate the tissue distribution and tumor targeting capability of DF-HSA in pancreatic carcinoma MIA PaCa-2 xenograft-bearing mice. As shown in Figure 6A, the images of Dylight 680-labeled DF-HSA showed fast tumor localization and accumulation after 2 h in MIA PaCa-2 xenograft. The strongest fluorescence intensity appeared in 24 h. Then the tumor-located image clearly maintained for about one week. There is no obvious fluorescence enrichment except the tumor *in vivo*. And with the increase of the dose, the fluorescence intensity of the tumor site was enhanced. For *ex vivo* imaging, the tumor and various organs were excised to further observe the distribution of DF-HSA. As Figure 6B revealed, there was no detectable signal observed in the tested organs of mice treated with DF-HSA while high fluorescence intensity was found in the tumor. Therefore, the imaging confirmed the specific distribution of DF-HSA with a prominent and lasting accumulation in the pancreatic carcinoma MIA PaCa-2 xenograft.

### *In vivo* therapeutic efficacy of DF-HSA

**Figure 7:**
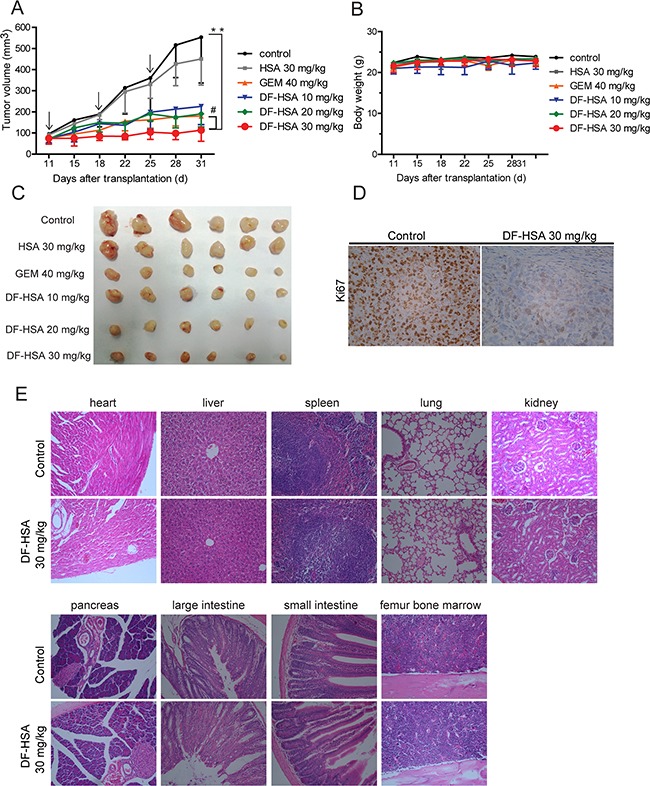
Therapeutic efficacy of DF-HSA against pancreatic carcinoma MIA PaCa-2 xenograft in athymic mice **A.** Tumor growing curves of human pancreatic carcinoma MIA PaCa-2 (n = 6). **, P ≤ 0.01, compared with the control; #, P ≤ 0.05, compared with the GEM group. Arrows denote the days of injection. **B.** Body weight change of MIA PaCa-2 xenograft-bearing mice. **C.** Photographs of the excised MIA PaCa-2 tumors from all groups of mice including those treated with HSA, DF-HSA and GEM and the control (n = 6) after sacrifice at 31 days. **D.** Effect of DF-HSA on tumor proliferation marker Ki-67 expression on paraffin-embedded MIA PaCa-2 was examined by immunohistochemistry (×400). **E.** Histopathological examination of various organs (H & E staining, ×200) of MIA PaCa-2 xenograft-bearing mice treated with DF-HSA at dosage of 30 mg/kg. No toxicopathological changes were found in the heart, liver, spleen, lung, kidney, pancreas, large intestine, small intestine and femur bone marrow.

Therapeutic experiments were performed with human pancreatic carcinoma MIA PaCa-2 xenograft in athymic mice. DF-HSA was administered by intravenous injection, once a week, for a total of 3 doses. As shown in Figure 7A and 7C, DF-HSA markedly inhibited the growth of MIA PaCa-2 xenograft tumor in a dose-dependent manner, while HSA showed no appreciable effect. In the end of the experiment, DF-HSA at doses of 10 mg/kg, 20 mg/kg and 30 mg/kg inhibited the growth of MIA PaCa-2 xenograft by 56.6%, 68.9% and 81.2%, respectively; while 40 mg/kg gemcitabine (GEM) inhibited tumor growth by 69.1%. In addition, there were no deaths and no significant body weight changes in mice of all treated groups (Figure 7B). By histopathological examination, no toxico-pathological changes were found in the heart, liver, spleen, lung, kidney, pancreas, large intestine, small intestine and femur bone marrow of mice treated with DF-HSA at dosage of 30 mg/kg (Figure 7E). These results suggested that the DF-HSA was highly effective against pancreatic carcinoma xenograft at well tolerated dosages.

Immunohistochemical staining data from treatment and control groups of tumor-bearing mice were compared to evaluate possible effects of DF-HSA on the proliferation of tumor cells *in vivo*. As shown in Figure 7D, strong immunohistochemical staining for cell proliferation index Ki-67 was observed in tumor tissue from the control group, whereas weak and moderate staining was found in that from the DF-HSA treated group. The results indicated that DF-HSA treatment exerted a significant anti-proliferation effect on tumor cells *in vivo*.

## DISCUSSION

Macropinocytosis which mediates the hyperactive influx of large endosomal vesicles is a highly important process in tumor growth and proliferation [31]. As reported, the mutated Ras or Src genes have been implicated in tumourigenesis and their transformed cells can stimulate macropinocytosis which serves as an important route for nutrient uptake in tumors. Indeed, the three proto-oncogenes (H-Ras, N-Ras, and K-Ras) of Ras family are the most frequently mutated oncogenes in cancer, with K-Ras being the most prevalent and potent to lead actin-mediated membrane ruffling and macropinosome formation [6, 32, 33]. Activating mutations of K-Ras gene are found in nearly 90% of pancreatic cancers [7, 34]. It is reported that K-Ras-transformed human pancreatic adenocarcinoma-derived MIA PaCa-2 cells (homozygous for the KRASG12C allele) display appreciably higher levels of macropinocytosis compared to wild-type K-Ras expressing BxPC-3 cells. Furthermore, the mutant MIA PaCa-2 cells exploit macropinocytosis to internalize extracellular albumin to support growth by degrading albumin into glutamine and other free amino acids, which can then enter the TCA cycle to sustain proliferation [1, 32]. The macropinocytosis inhibitor EIPA can significantly reduce the uptake of a large amount of albumin and inhibit tumor xenograft growth of pancreatic carcinoma MIA PaCa-2 cells that display intensive macropinocytosis. By contrast, pancreatic carcinoma BxPC-3 cells which show wild-type Ras expression took only little albumin and EIPA had no effect on the growth rate of this low-level-macropinocytosis BxPC-3 tumor [1]. Therefore, albumin may be able to serve as a carrier for macropinocytosis-mediated targeting therapy. In this study, pancreatic cancer MIA PaCa-2 cells showed more efficient micropinocytosis-mediated cellular uptake of human serum albumin, compared with wild-type K-Ras-expressing BxPC-3 cells; this is in conformity with that of previous report [1]. Furthermore, albumin-integrated β-defensin DF-HSA, a newly constructed and prepared bioactive protein in our laboratory, has definitely displayed intensive macropinocytic entry in pancreatic carcinoma MIA PaCa-2 cells; and the uptake intensity was much higher in MIA PaCa-2 cells than that in BxPC-3 cells. In addition, fluorescent signals of DF-HSA and TMR-dextran taken up by MIA PaCa-2 cells were highly co-localised inside the cells; the uptake of DF-HSA was obviously inhibited by EIPA, the macropinocytosis specific inhibitor, in a concentration-dependent manner. Thus, these results demonstrate that the intracellular entry of DF-HSA is apparently mediated by macropinocytosis and there exists a differential pattern of intensity between K-Ras mutant cancer cells and wild-type expressing cancer cells. Further studies of *in vivo* imaging demonstrate that DF-HSA can selectively accumulate in MIA PaCa-2 tumor location and retain for a long period of time. Accordingly, it can be concluded that the strategy of utilizing albumin as the carrier for macropinocytosis-mediated targeting therapy is feasible.

Recently, there is growing evidence that defensins can be used as potential agents in cancer treatment [17, 35, 36]. In the human innate immunity, defensins play an important role as antimicrobial peptides [37]. It is attractive that defensins are more prone to interact with tumor cells rather than normal cells, resulting in disruption of membranes; because the cationic property of defensins enables them to interact with the cancer cells of which the membrane largely presents various anionic molecules such as anionic phosphatidylserine and sialic acid residues [17, 18]. Various expression levels of β-defensin 2 (HBD2) have been found in the epithelial layer of many human organs, it exhibits a significantly disrupting membrane (cytoplasmic and/or mitochondrial) activity against cancer cells while the threshold called minimum inhibitory concentration was reached [10, 38–40]. As endogenous peptides, defensins have a great potential in the field of cancer therapy. For the development of defensin-based antitumor therapeutics, we adopted a macropinocytosis-mediated strategy that renders massive defensins entering into the cell and acting intracellularly, namely, macropinocytosis-mediated targeting therapy. In the present study, we constructed an albumin-integrated defensin (DF-HSA) comprising HBD2 and HSA for which HBD2 was used as the “effector” molecule and HSA as the macropinocytosis mediator. Because HBD2 contains three disulfide bonds and can kill microorganisms as an antimicrobial peptide, HBD2 with biological functions is difficult to produce directly in *Escherichia coli* expression system [41, 42], we employed *Pichia pastoris* secretory expression system to produce DF-HSA. The study shows that newly designed and genetically engineered albumin-integrated β-defensin DF-HSA is characterized with several attributes. Firstly, DF-HSA displays intensive macropinocytosis-mediated process, leading to a massive uptake in cancer cells. Furthermore, the uptake intensity is much higher in K-Ras mutant MIA PaCa-2 pancreatic cancer cells than that in wild-type K-Ras expressing BxPC-3 cells. Secondly, DF-HSA exerts much more potent cytotoxicity to cancer cells than the corresponding free β-defensin HBD2. This differential pattern in cytotoxicity correlates well with the differential patterns of uptake. Thirdly, DF-HSA has a highly specific biodistribution, exhibiting clear and long-lasting localization by imaging. Fourthly, of particular importance, DF-HSA exerts potent therapeutic efficacy against the K-Ras mutant MIA PaCa-2 pancreatic carcinoma xenograft in athymic mice. It can markedly suppress tumor growth at well tolerated doses. The postulated reason to explain the high effectiveness of DF-HSA against tumor growth was that the albumin-integrated β-defensin can accumulate massively in tumor cells through macropinocytosis-mediated process and exert antitumor effect efficiently and intracellularly in cancer cells, especially the K-Ras mutant pancreatic cancer cell.

The study result highlights the differential pattern of DF-HSA action between the K-Ras mutant cancer cells and wild-type expressing cancer cells. As shown, the proliferation and colony formation capability of MIA PaCa-2 cells was markedly inhibited, while little effect was found in BxPC-3 cells with the same treatment. This is in conformity with the pattern of uptake intensity, indicating that MIA PaCa-2 cells have higher levels of DF-HSA uptake than BxPC-3 cells. However, the free β-defensin HBD2 only had weak cytotoxicity to MIA PaCa-2 cells and BxPC-3 cells. This study has shown that the albumin-integrated β-defensin DF-HSA is significantly different from the free β-defensin HBD2. As reported, the antitumor activity of human defensins can be completely abolished or reduced by low levels of serum for the reason that the serum components, such as albumin and low-density lipoprotein, are acting as potent inhibitors of peptide-mediated cell lysis [17, 43, 44]; apparently, in the case of free defensin, albumin may reduce its activity and this may pose as a restrictive factor in the use of free defensins *in vivo*. However, in the case of albumin-integrated defensin, the integrated albumin did not reduce the activity of defensin; by contrast, enhanced the latter's activity. The designed and constructed albumin-integrated defensin DF-HSA has two major advantages. Firstly, DF-HSA can overcome serum inactivation because HBD2 is brought into the tumor cells in the albumin-integrated defensin format by macropinocytosis, evading the interference of serum. Secondly, DF-HSA shows specific effect against in K-Ras mutant MIA PaCa-2 cells, in which large amounts of DF-HSA can be internalized by micropinocytosis; however, those normal cells which lack macropinocytic activity would internalize less amount of DF-HSA.

Previous reported that if internalized inside eukaryotic cells, defensins can induce the permeation and swelling of mitochondria via mitochondrial membrane disruption, which causes the release of cytochrome C, leading to apoptosis [17, 45–47]. The present study revealed that DF-HSA can damage the mitochondria in MIA PaCa-2 cells and induce cancer cell apoptosis by mitochondrial pathway; however, DF-HSA only had little influence on BxPC-3 mitochondria. These results collectively suggest that after DF-HSA in the albumin-integrated format was transported into MIA PaCa-2 cells through macropinocytosis, leading to triggering apoptosis via mitochondrial membrane damage.

In summary, defensins, the major components of innate immune system, are reported to be potential effective agents for cancer therapy. According to the strategy for macropinocytosis-mediated targeting cancer therapy, a recombinant macropinocytosis-oriented tailor-made β-defensin DF-HSA has been prepared and evaluated. The cytotoxicity of DF-HSA to cancer cells was more potent than that of relevant free β-defensin. DF-HSA displayed intensive macropinocytosis-mediated uptake in K-Ras mutant pancreatic carcinoma MIA PaCa-2 cells; and the intensity was much higher than that in wild type K-Ras expressing pancreatic carcinoma BxPC-3 cells. Compared in terms of IC50 values, MIA PaCa-2 cells were more sensitive to DF-HSA than the BxPC-3 cells. By imaging DF-HSA showed specific biodistribution with prominant and prolonged tumor localization. Notably, DF-HSA exerted highly therapeutic efficacy against the K-Ras mutant pancreatic carcinoma MIA PaCa-2 xenograft in athymic mice. To our knowledge, this is the first report to design a new format of defensin with intensive macropinocytosis-mediated internalization and confirm its therapeutic efficacy. The study suggests that the recombinantly optimized β-defensin DF-HSA could be a promising agent in macropinocytosis-mediated targeting cancer therapy, in particular, for K-Ras mutant cancers.

## MATERIALS AND METHODS

### Cell lines and culture conditions

Human pancreatic carcinoma cell line MIA PaCa-2 (K-Ras mutant cells), human lung cancer cell lines A549 and H460 were purchased from the American Type Culture Collection (ATCC), these cell lines were tested by STR analysis by China Center For Type Culture Collection (CCTCC) in 2013. The human pancreatic carcinoma BxPC-3 cell line (wild-type K-Ras expressing cells) was provided by the Cell Resource Center, Institute of Basic Medical Sciences, CAMS/PUMC (which is the headquarter of National Infrastructure of Cell Line Resource, National Sciences and Technology Infrastructure) in July, 2014; the identity of the cell line was authenticated with STR profiling and used within 6 months. Human hepatocyte cell line L02 was a kind gift from Professor Hong-wei He (Institute of Medicinal Biotechnology, Chinese Academy of Medical Sciences and Perking Union Medical College). MIA PaCa-2 cell line was cultured in Dulbecco's modified Eagle medium (Hyclone; Thermo Fisher Scientific). Other cell lines were cultured in RPMI-1640 (Hyclone; Thermo Fisher Scientific) medium; in addition, 1 mM sodium pyruvate (Gibco) was supplemented for BxPC-3 cell line. All the media were supplemented with 10% (v/v) of heat-inactivated fetal bovine serum (FBS, Gibco; Life Technologies), penicillin G (100 U/mL), and streptomycin (100 μg/mL). The cells were cultured in an incubator, maintained at 37°C with 5% CO_2_.

### Construction of expression vectors

As shown in Figure 1A, the full gene fragments of the albumin-integrated β-defensins DF-HSA and HSA-DF mainly consist of the gene encoding HBD2 (41 amino acids), HSA (585 amino acids), and the linker peptide (G4S)_2_. The artificial gene encoding for HBD2 was synthesized by Invitrogen Company and cloned into the pUC57s vector. The vector pHBM-HSA carrying the gene encoding for HSA was constructed in this laboratory. After three rounds of PCR and DNA cloning process, the resultant 1908 bp fragments were digested by *SnaB I/Not I*, inserted into expression vector to generate expression plasmids pHBM-DF-HSA and pHBM-HSA-DF, respectively. DNA sequencing determination was accomplished by the method provided by Invitrogen Corp.

### Expression and purification of albumin-integrated defensins

The sequence-verified expression plasmids pHBM-DF-HSA and pHBM-HSA-DF were linearized by *Sal I*, used for *Pichia pastoris* strain GS115 electro transformation to produce the recombinant proteins, respectively. The procedures were according to previously reported method [48]. The expressed fusion proteins were mainly secreted to the supernatant. Then the proteins were purified by affinity chromatography through a C-terminal 6×His-tag (His Trap HP, GE Healthcare) according to the manufacturer's protocol. The purified proteins were analyzed by SDS-polyacrylamide gel electrophoresis (SDS-PAGE) and the protein concentration was determined by the BCA protein assay kit (Thermo Fisher Scientific, USA).

### Confocal analysis for internalization

Pancreatic carcinoma MIA PaCa-2 and BxPC-3 cells were separately cultured on the eight-well chamber slide (Lab TakChamber Slide System, Nunc). Macropinosomes were marked using a high-molecular-mass TMR-dextran (MW 7,000, Invitrogen) uptake assay. After cell attachment, the FITC-labeled HSA, FITC-labeled DF-HSA or/and TMR-dextran were added at a final concentration of 1 mg/ml and incubated for another 0 to 5 h at 37°C. The experimental operation of FITC labeling protein was performed as the described protocol [49]. At the end of the incubation period, cells were washed three times in cold PBS and then immediately fixed in 4% paraformaldehyde for 15 min at room temperature. DAPI (Zhongshan Golden Bridge Biotechnology) was used to stain the nuclei. Images were captured by the laser scanning confocal microscope to examine the DF-HSA uptake capability of pancreatic carcinoma cells and the data were analyzed with the Imagepro plus software.

### Cytotoxicity assay

The cytotoxicity determination was performed using MTT assay. HBD2 was purchased from Abcam. Cells were seeded in 96-well plates and after 24 h, were treated with different concentrations of tested agents (DF-HSA, HSA, HBD2 or HSA-DF) for 48 h. After that, 20 μL of MTT (Amresco) solution (5 mg/mL) were added to each well and 4 h later, the supernatant was removed and 150 μL DMSO were added to each well on a shaker for 10 min. The absorbance was measured at 570 nm using a microplate reader (Thermo Fisher Scientific, USA). Cells treated with PBS served as control. The 50% inhibitory concentration (IC50) of the tested samples was calculated.

### Clonogenic assay

Clonogenic assay was performed to detect cell colony formation capability. Cells were trypsinized and seeded in triplicates at a density of 50-200 cells/well in 96 well plate in 200 μl of medium containing 10% FBS. After incubated at 37°C with 5% CO2 for 24 h, various concentrations of tested agents (DF-HSA and HSA) were added respectively and further grown for 10-15 days. Colonies were counted in an inverted microscope. The colony formation capability was calculated by dividing the number of colonies of treated cells by that of the control.

### Flow cytometry for apoptosis analysis

Flow cytometry was applied to analyze the influence of the albumin-integrated defensin on MIA PaCa2 and BxPC-3 pancreatic cancer cells. Cells were plated in 12-well culture plates at the density 1 × 10^5^ cells per well and incubated at 37°C for 24 h. Then different concentrations of the tested samples (DF-HSA, HBD2 and HSA) were added for another 24 h, respectively, while cells treated only with the medium were used as control. Following the instructions provided by the manufacturer of Annexin V-FITC Apoptosis Kit (Solarbio, China), cells were resuspended in Annexin V binding buffer; and then Annexin V-FITC and propidium iodide (PI) were added and incubated for 30 min at 4°C. The fluorescence-labeled cells were analyzed with FACS Calibur (BD Company).

### Western blot

Protein extracts were prepared with the ice-cold high efficiency RIPA tissue/cell lysis buffer (Beijing Solarbio Science & Technology Co., Ltd). Thirty micrograms of each total protein were applied on 12% SDS-PAGE, then subjected to electrophoretic analysis and blotting. All primary antibodies were incubated overnight at 4°C; and then incubated with goat/rabbit anti-mouse/rabbit/goat peroxidase-coupled antibody (Zhongshan Golden Bridge Biotechnology, diluted 1:5,000) at RT for 2 h which was used for detecting the primary antibody binding. Protein bands were visualized with an enhanced chemiluminescence kit (Merck Millipore).

For Western blot analysis, the following primary antibodies were used: anti-β-actin (Cell Signaling Technology, #3700) as loading controls; anti-caspase-3 (Cell Signaling Technology, #9662), anti-cleaved caspase-3 (Cell Signaling Technology, #9661), anti-bax (Cell Signaling Technology, #5023), anti-bcl-2 (Cell Signaling Technology, #2870), anti-cytochrome C (anti-Cyt-c, Cell Signaling Technology, #4280) and anti-caspase-9 (Cell Signaling Technology, #9502), anti-P53 (Cell Signaling Technology, #9282), anti-HBD2 (Santa Cruz Biotechnology, #sc-10854) and anti-His-Taq (Abmart, #M20001). The levels of the specific protein bands were all determined following the same procedures that were described above.

### Transmission electron microscopy observation

Transmission electron microscopy was applied to investigate the subcellular changes in MIA PaCa-2 cells after exposing to DF-HSA. The ultrastructural changes of the treated cells were compared with that of control. DF-HSA treated cells were collected and washed with PBS twice and fixed in 2.5% glutaraldehyde at 4°C for 2 h, then rinsed in PBS, and embedded in 3% agarose. The following procedure included fixation, dehydration, imbedding, polymerization, ultrathin section and staining. The sections were analyzed by using transmission electron microscope (Tecnai G2 Spirit, FEI).

### Determination of the effect of DF-HSA on mitochondrial membrane potential

Mitochondrial potential sensor JC-1 (Sigma) was used to measure the alteration of the mitochondrial membrane potential. Human pancreatic carcinoma MIA PaCa-2 cells were seeded at a density of 1 × 10^5^ cells/well in the 12-well culture plates and incubated at 37°C overnight for cell attachment. The cells were then treated with different concentrations of tested samples (DF-HSA, HBD2 and HSA) for 24 h, while cells treated with the medium alone served as control. Following that, final concentration of JC-1 (5 μg/ml) was added into the culture medium and incubated for 10 min at 37°C in the dark. Next, cells were trypsinized and washed in PBS. Stained cells were examined with FACS Calibur (BD Company).

### Determination of reactive oxygen species (ROS)

The level of intracellular ROS was measured using CM-H2DCFDA (Invitrogen). Briefly, 1 × 10^5^ MIA PaCa-2 cells/well were plated in 12-well plates and allowed to attach overnight. Then, cells were treated with 10 μM CM-H2DCFDA for 30 min at 37°C. Subsequently, the cells were washed with PBS and treated with different concentrations of tested samples (DF-HSA, HBD2 and HSA) in 500 μL solution. The levels of intracellular ROS were analyzed using FACS Calibur (BD Company).

### *In vivo* and *ex vivo* optical imaging of fluorescein-labeled DF-HSA

For imaging experiment, DF-HSA was labeled with the fluorescein DyLight 680 according to the manufacturer's instruction (DyLight 680 Antibody Labeling Kit, Thermo). The tumor-targeting capability of DF-HSA was investigated using MIA PaCa-2 xenograft model. Cells were inoculated to the right armpit of athymic mice. When the solid tumors reached a volume of about 100-400 mm^3^, DyLight 680 labeled DF-HSA was injected into the tail vein of mice (n = 3) at dosages of 10, 30, and 50 mg/kg. Next, the mice were anesthetized by isofluorane at several selected time points and placed in the imaging chamber of an IVIS-200 system (Xenogen) for observation. Images and measurements of DyLight 680 fluorescence signals were analyzed using Living Image-software (Xenogen). At 120 h, the animals were euthanized, specimens were taken from the tumors and various organs for *ex vivo* fluorescence imaging.

### *In vivo* therapeutic efficacy

For evaluation of the therapeutic efficacy of DF-HSA, the animal experiment was performed with the pancreatic carcinoma MIA PaCa-2 xenograft model. The study protocols were in accordance with the regulations of Good Laboratory Practice for Non-clinical laboratory studies of drugs issued by the National Scientific and Technologic Committee of People's Republic of China. Female BALB/c athymic mice (6-8 weeks old) were purchased from the Center of Experimental Animals, Academy of Military Medical Sciences in Beijing. MIA PaCa-2 cells suspended in PBS (1 × 10^7^ cells/200 μl) were inoculated subcutaneously in right armpit of athymic mice. Approximately one month later, the grown tumors were taken out from the nude mice and dissected aseptically in the sterile saline. The pieces of tumor tissue (2 mm^3^ in size) were then transplanted into the right armpit of nude mice by a trocar, and the wound was sealed by celloidin. Tumor-bearing mice were randomly divided into 6 groups (n = 6) and treated respectively with DF-HSA (i.v.), HSA (i.v.), and gemcitabine (GEM, i.p.), once a week, a total of three injections. Physiologic saline was injected as controls. GEM as positive control was given at dose of 40 mg/kg; HSA was given at dose of 30 mg/kg; and DF-HSA was given at doses of 10, 20, and 30 mg/kg, respectively. Tumor size was measured every 3 - 4 days and tumor volume was calculated with the following formula: V (mm^3^) = 0.5×*a*×*b*^2^, where *a*, and *b* represent the long and the perpendicular short diameters of the tumor, respectively.

At the end of the experiment, the mice were sacrificed and the tumors were dissected, weighed, and photographed. The inhibition rate of tumor growth was calculated as [1 − weight (treated)/tumor weight (control)] × 100%. In addition, specimens were taken from the tumor and various organs for histopathological examination. The specimens were fixed in 10% formalin and embedded in paraffin. Sections were cut into 5 μm in thickness and stained with hematoxylin and eosin (H & E). Histopathological changes were observed with a Leica microscope system.

### Immunohistochemical analyses

Immunohistochemical analyses were performed on tumor tissue sections using antibody specific for Ki-67 as described previously [50]. Stained sections were observed using a microscope, and the images were analyzed using the Leica software system.

### Statistical analysis

Data were calculated with Microsoft Excel or GraphPad Prism 6 software, which are presented as the mean ± SD. One-way ANOVA followed by Student's t-test was used to determine significant differences between two groups of data. P values < 0.05 were considered statistically significant.
